# Transcriptome Sequencing and Mass Spectrometry Reveal Genes Involved in the Non-mendelian Inheritance-Mediated Feather Growth Rate in Chicken

**DOI:** 10.1007/s10528-023-10643-y

**Published:** 2024-01-27

**Authors:** Mohan Qiu, Zengrong Zhang, Shiliang Zhu, Siyang Liu, Han Peng, Xia Xiong, Jialei Chen, Chenming Hu, Li Yang, Xiaoyan Song, Bo Xia, Chunlin Yu, Chaowu Yang

**Affiliations:** Animal Breeding and Genetics Key Laboratory of Sichuan Province, Sichuan Animal Science Academy, 7# Niusha Road, Chengdu, 610066 Sichuan China

**Keywords:** Differentially expressed genes, Feather growth rate, RNA sequencing, Mass spectrometry, Chicken

## Abstract

The feather growth rate in chickens included early and late feathering. We attempted to characterize the genes and pathways associated with the feather growth rate in chickens that are not in agreement with Mendelian inheritance. Gene expression profiles in the hair follicle tissues of late-feathering cocks (LC), early-feathering cocks (EC), late-feathering hens (LH), and early-feathering hens (EH) were acquired using RNA sequencing (RNA-seq), mass spectrometry (MS), and quantitative reverse transcription PCR (qRT‑PCR). A total of 188 differentially expressed genes (DEGs) were ascertained in EC vs. LC and 538 DEGs were identified in EH vs. LH. We observed that 14 up-regulated genes and 9 down-regulated genes were screened both in EC vs. LC and EH vs. LH. MS revealed that 41 and 138 differentially expressed proteins (DEPs) were screened out in EC vs. LC and EH vs. LH, respectively. Moreover, these DEGs and DEPs were enriched in multiple feather-related pathways, including JAK-STAT, MAPK, WNT, TGF-β, and calcium signaling pathways. qRT-PCR assay showed that the expression of WNT8A was decreased in LC compared with EC, while ALK and GRM4 expression were significantly up-regulated in EH relative to LH. This study helps to elucidate the potential mechanism of the feather growth rate in chickens that do not conform to genetic law.

**Supplementary Information:**

The online version contains supplementary material available at 10.1007/s10528-023-10643-y.

## Introduction

In contemporary chicken production, sex identification could be performed on the basis of the feathering rate, a noninvasive approach to identify hens and cocks (Derks et al. 2018). The feathering rate phenotype which is usually observed in chickens within 24 h is related to sex, including early feathering and late feathering (Fang et al. 2018). The sex chromosome composition of cocks is ZZ and that of hens is ZW (Lin et al. 2021). Evidence showed that the K locus occupied on the chromosome Z and was associated with the late-feathering phenotype, whereas the k + allele was related to the early-feathering phenotype (Derks et al. 2018; Okamura et al. 2019). Theoretically, the descendant of late-feathering cocks (LC) and early-feathering hens (EH) should be LC and late-feathering hens (LH). Nevertheless, in the practical production of commercial chickens, there are still EH and early-feathering cocks (EC) with 7–10% probability. It is supposed that feather growth rate may be connected with autosomal inheritance and downstream gene modulation.

The evolution of the feather follicle, which is characterized by intricate physiological processes manifested in the morphology, is influenced and regulated by several factors (Wang et al. 2022; Xu et al. 2022). Wingless/integrated (Wnt) pathway components are typically specialized proteins that have appeared along with the Wnt signaling cascade during evolution and are considered to possess important functions in the Wnt cascade (Zhao et al. 2022). For example, cell proliferation, survival, differentiation, and embryonic development could be defined by the Wnt/β–catenin pathway (Tewari et al. 2021). Of note, the Wnt/β–catenin signaling pathway, an evolutionarily conserved pathway, functions in feather follicles (Chen et al. 2020; Tewari et al. 2021). Additionally, the transforming growth factor-β (TGF-β) pathway, a convergent signaling node, regulates gene expression, mitosis, metabolic growth, motility, survival, apoptosis, differentiation, and feather growth rate (Kahata et al. 2018; Hariyanto et al. 2021). However, the signaling pathways that regulate feather growth require further study. Particularly, the feather growth rate-associated genes and signaling pathways that do not conform to genetic laws have not yet been clarified.

To determine feather growth rate-related mechanisms in chickens that are inconsistent with the Mendelian inheritance law, we integrated transcriptome and proteome to analyze the differentially expressed genes (DEGs) and proteins (DEPs) in EC vs. LC and EH vs. LH. Moreover, the function and pathway were analyzed. Quantitative reverse transcription PCR (qRT-PCR) was used to verify the DEGs. For the first time, we showed the genes related to feather growth rate that did not conform to the Mendelian inheritance law, which may provide a new theoretical basis for chicken feather growth rate.

## Materials and Methods

### Sample Collection

All experiments were performed on specific pathogen-free White Feather chickens and the Sichuan Dahen Animal breeding company provided 1-day-old chicks. Hair follicle tissues were acquired from LC, LH, EH, and EC (*n* = 3).

### Transcriptome Sequencing

To extract the total RNA from the hair follicle samples, TRIzol reagent (Invitrogen, Carlsbad, CA, USA) was utilized according to the manufacturer’s instructions. Subsequently, RNA integrity, purity, and concentration were estimated using 1% agarose gels and spectrophotometer (Thermo Fisher Scientific, Inc., Waltham, MA, USA). First, an EpicentreRibo-Zero™ rRNA Depletion Kit (Epicentre, WI, USA) was employed to remove ribosomal RNA. Subsequently, RNA sequencing (RNA-seq) libraries were generated using the NEB Ultra™ Directional RNA Library Prep kit (New England BioLabs, Inc.). Moreover, the libraries were sequenced on Hiseq X Ten (Illumina, San Diego, CA, USA). The Fast-QC (http://www.bioinformatics.babraham.ac.uk/projects/fastqc/) software was employed to evaluate the overall quality of sequencing data. The HISAT2 software was used to compare clean reads with the reference genome GRCg6a. The DEGs were screened by utilizing DESeq2 on the basis of |log2(Fold change)|> 1 and *P* value < 0.05. Gene ontology (GO) and Kyoto Encyclopedia of Genes and Genomes (KEGG) enrichment analyses were utilized to analyze the function and pathways of DEGs.

### Sample Preparation and Liquid Chromatography Tandem Mass Spectrometry (LC–MS/MS) Analysis

Proteins from the hair follicle tissues were extracted using SDT lysis buffer. To quantify the protein level, the BCA protein assay kit (Bio-Rad, USA) was used. Protein digestion by trypsin was conducted in accordance with the filter-aided sample preparation procedure as previously described (Wisniewski et al. 2009). The digest peptides were desalted on C18 Cartridges, concentrated by vacuum centrifugation, and reconstituted in 40 µL of 0.1% (v/v) formic acid. Subsequently, peptide mixtures were labeled by utilizing iTRAQ reagent and TMT reagent following the manufacturer’s instructions. To fractionate the labeled peptides, the High pH Reversed-Phase Peptide Fractionation Kit (Thermo Scientific) was employed. The acquired fractions were desalted on C18 Cartridges and subsequently concentrated by vacuum centrifugation.

LC–MS/MS analysis was performed using Q-Exactive™ mass spectrometer (Thermo Scientific) for 1–1.5 h. The samples were added to an inverting trap column connected to the C18 reversed-phase analytical column by utilizing buffer A and segregated by linear gradient using buffer B (300 mL /min). A data-dependent Top10 way was applied to obtain MS data, which dynamically selects the most rich parent ions from survey scans (300–1800 m/z) for HCD fragmentation. The AGC target was set to 3e6, and the injection time at maximum was 10 ms. The dynamic exclusion time was 40.0 s. The measurement scan and HCD spectral resolution were set to 70,000 resolution at 200 m/z, and 17,500 at 200 m/z, respectively, and the isolation width was 2 m/z. The normalized collision energy was defined as 30 eV, and the underfill ratio was set as 0.1%. The mass spectrometer operates in the peptide recognition mode. All raw data have been deposited in iProX database with accession number: IPX0007552000.

### Bioinformatics Analysis

The Proteome Discoverer 1.4 software was used to quantify the peptides in the MS raw data. A Mascot 2.2 software was used to correlate the MS/MS spectral data with the UNIPROT protein database to determine *Gallus gallus*. The screening of the DEPs was performed on the basis of Fold Change > 1.2 and *P* value < 0.05. CELLO and the InterProScan software were utilized to analyze the subcellular localization of proteins and identify protein domain signatures, respectively. The NCBI BLAST + client software and InterProScan were adopted to analyze homolog sequences for identification of DEP sequencing. Later on, GO annotation was implemented via the software program Blast2GO, the results of which were visualized by the R-tool. Subsequently, the analysis of GO terms and KEGG pathways of DEGs was fulfilled using KOBAS 2.0 server.

### qRT-PCR

The total RNA was isolated using TRIzol reagent (Invitrogen, USA) following the manufacturer’s instructions. To synthesize complementary DNA, a Reverse Transcription Kit (Applied Biosystems, USA) was used. Subsequently, qRT-PCR was performed using SYBR Green Master Mix (Roche Diagnostics, UK). The relative expression of WNT8A, CHRM2, CCKBR, FGF22, CD109, GHRL, ALK, GRM4, C5, and NTF3 was analyzed by employing the 2^−ΔΔCt^ method with GAPDH as the normalizer. The oligonucleotide primers for target genes are presented in Table 1.

**Table 1:** The primer sequences

Gene	Primer sequence (5′ to 3′)	Amplified fragment (bp)
gga-WNT8A-F	CAGCGACAACGTGGAGTTTG	108
gga-WNT8A-R	CCGACTTCGTTGTTGTGCAG	
gga-CHRM2-F	GGCAGGCATGATGATTGCAG	153
gga-CHRM2-R	GTGCCAAAAGTGACAGCAGG	
gga-CCKBR-F	TGAGGCAGGCATGGTATGTC	85
gga-CCKBR-R	GAGATGAGCCCGTAAGCCAC	
gga-FGF22-F	CAACTGCAAGTTCACGGAGC	115
gga-FGF22-R	CGGCCTCCCTTTGCTATTGA	
gga-CD109-F	TTCAGGCGGAGTTCCTCAAC	125
gga-CD109-R	CTCGAAAGGTTTCCCAGCCT	
gga-GHRL-F	AGATTACATCGCCGAGGCAC	164
gga-GHRL-R	AGCATCTTCTCCAGTGCTTGT	
gga-ALK-F	ACTGTGAGATGGACGAGTGC	109
gga-ALK-R	GGCTCAGCTATGCAAGACAC	
gga-GRM4-F	ATCATCCTCTTCCACCCGGA	172
gga-GRM4-R	TGGTGGACAGTGCTTGTGTT	
gga-C5-F	TGTTGCTTATTCTTCTGGCCA	101
gga-C5-R	CCTCTGGGCCAGCTAAATCT	
gga-NTF3-F	GTGAAATCAGACTTCCAGCCA	125
gga-NTF3-R	AGGTACAGTGGTGGTGGTTC	
gga-GAPDH-F	AGAAGGCTGGGGCTCATT	158
gga-GAPDH-R	TGCTAAGCAGTTGGTGGTG	

### Statistical Analyses

Statistical analyses were performed using Statistical Package for the Social Sciences (version 20, IBM, Armonk, NY, USA). Data were obtained from at least three independent samples and displayed as means ± standard deviations. Difference was ascertained by Student’s t test. A *P*-value of < 0.05 was considered statistically significant.

## Results

### General Information on RNA-seq Results

**Fig. 1:**
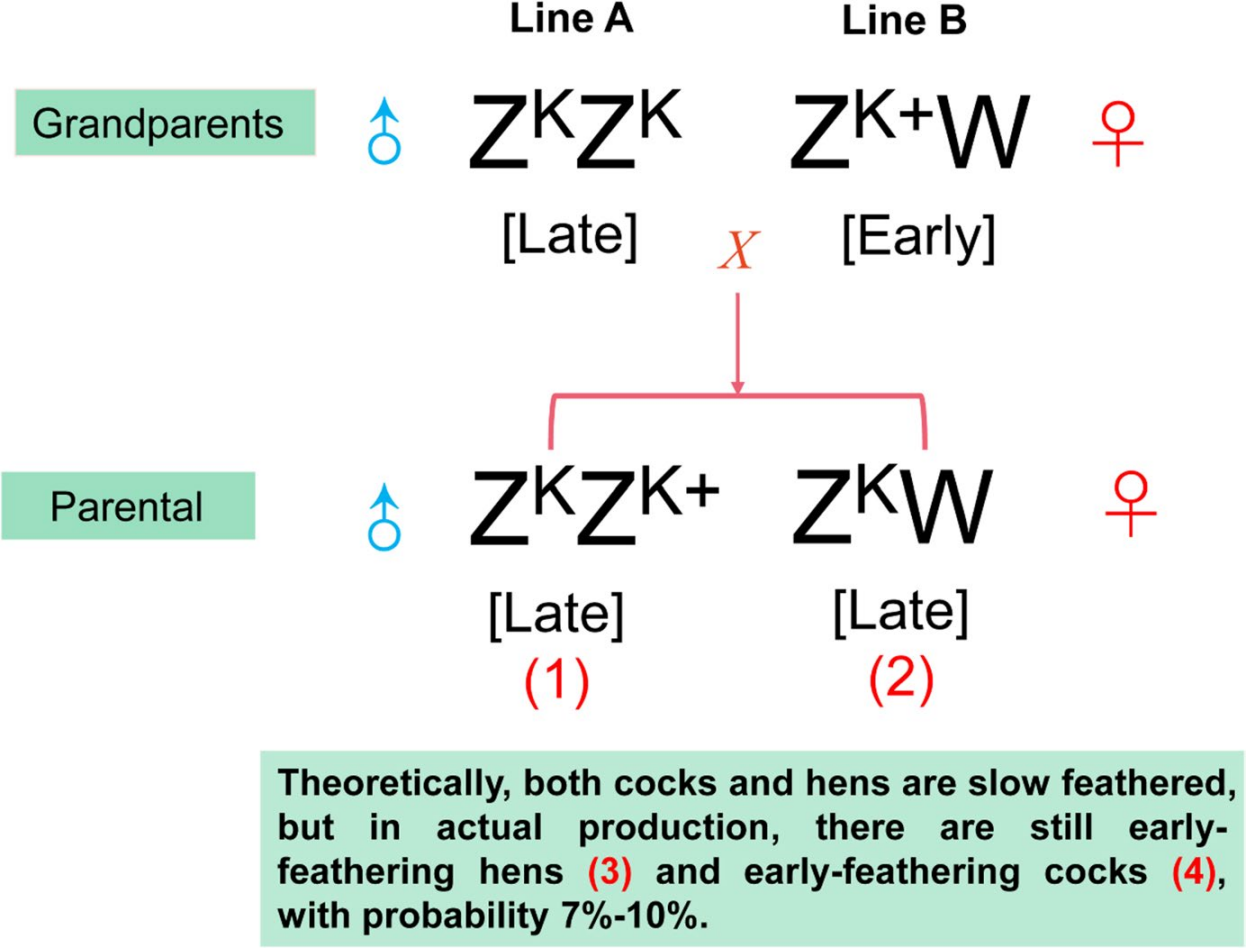
The genetic diagram of offspring from late-feathering cocks (LC) and early-feathering hens (EH). The K locus occupies the chromosome Z and is relevant with the late-feathering phenotype, whereas the k + allele is recessive and is associated with the early-feathering phenotype. According to the Mendelian inheritance law, the offspring of LC and EH should be LC and late-feathering hens (LH); however, early-feathering cocks (EC) and EH are found in actual production. The numbers (1–4) marked in the figure are the samples used in this study

To decipher potential mechanisms that were responsible for the feathering rate phenotype not conforming to the Mendelian inheritance law, we analyzed DEGs and DEPs in EC (not conform to the Mendelian inheritance law) vs. LC (conform to the Mendelian inheritance law) and EH (not conform to the Mendelian inheritance law) vs. LH (conform to the Mendelian inheritance law) (Fig. 1). Subsequently, RNA-seq was conducted using 12 samples, the results of which are presented in Table 2. As depicted in Supplemental Table 1, the mapping ratio for all the samples was more than 90%.

**Table 2:** Sequencing data statistics

Sample	TotalReads _Before	TotalBase _Before	TotalReads _After	TotalBase _After	BaseFilter %
EC1	84977206	12746259632	82142254	12311260545	0.965872413
EC2	96335978	14450029873	92945348	13929997321	0.964011662
EC3	114982860	17246995915	111334938	16687202536	0.967542557
EH1	103184602	15477304978	99298738	14881180317	0.961483949
EH2	113366086	17004479513	109381872	16394310132	0.964117139
EH3	119834564	17974736051	115499494	17311435074	0.963098152
LC1	91455302	13717949350	88531544	13269171758	0.967285373
LC2	109164960	16374327992	104494802	15660324810	0.956394963
LC3	126802534	19019906418	122760902	18399047763	0.967357429
LH1	91824178	13773280672	88844956	13315680125	0.966776213
LH2	101964918	15294352648	98824042	14812901304	0.968520973
LH2	115321506	17297793283	111477484	16707854547	0.965895145

### Identification of DEGs Between Late-Feathering Chickens that Conform to Genetic Laws and Early-Feathering Chickens that do not Conform to Genetic Laws

**Fig. 2:**
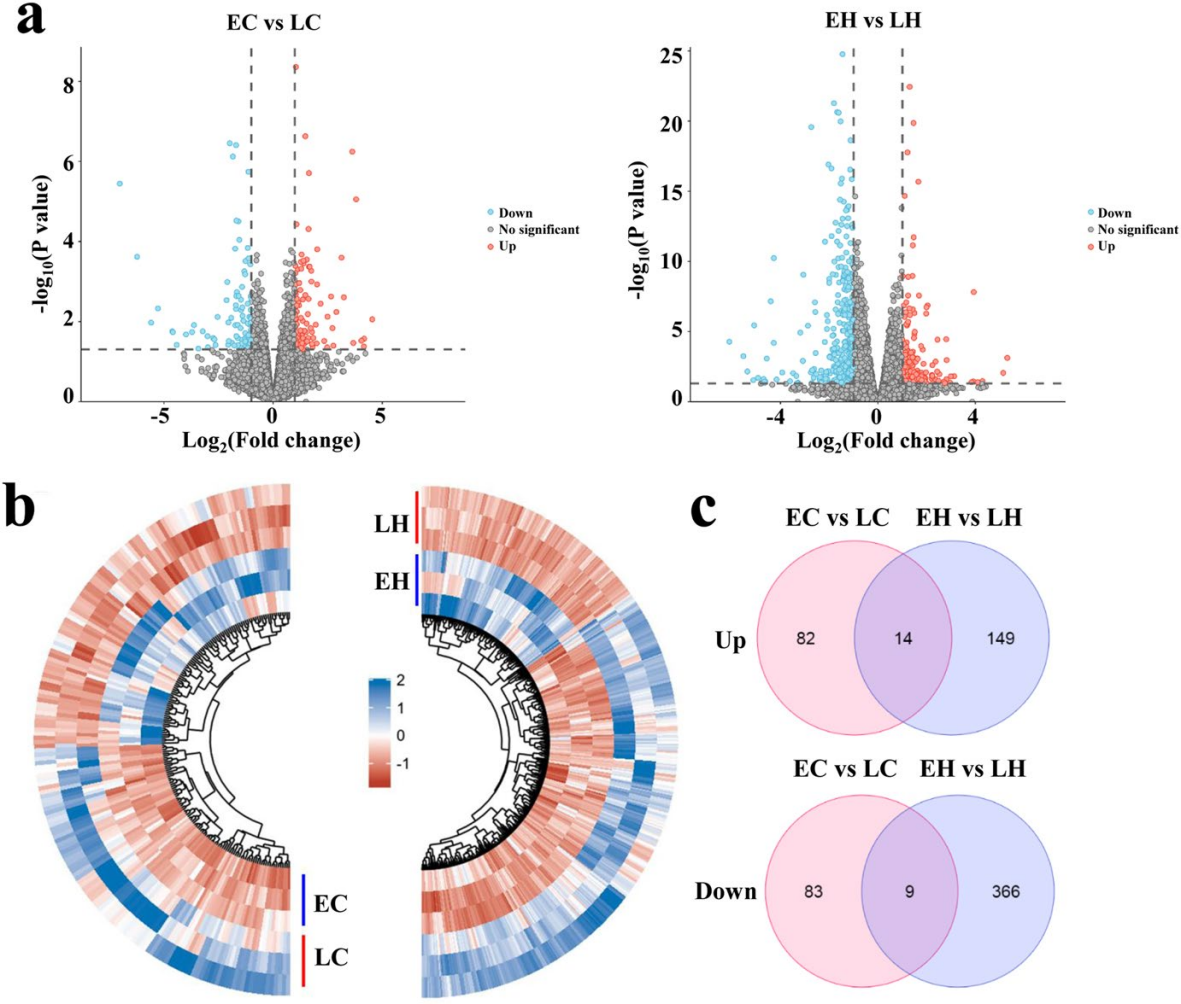
Differentially expressed genes (DEGs) identification in chickens obtained from RNA sequencing (RNA-seq). **a** Volcano plot visualizes the DEGs in early-feathering cocks (EC) vs. late-feathering cocks (LC) (left panel) and early-feathering hens (EH) vs. late-feathering hens (LH) (right panel) using RNA-seq (*n* = 3). **b** The hierarchical clustering heatmap of all the DEGs in EC vs. LC (left panel) and EH vs. LH (right panel). **c** The intersection results of the DEGs in EC vs. LC and EH vs. LH

To discriminate the potential genes that were implicated in chicken early-feathering and late-feathering phenotypes, a comparative analysis was conducted in chicken. We obtained 188 DEGs with 96 up-regulated and 92 down-regulated genes, respectively, in the EC group as opposed to the LC group (Fig. 2a, Supplemental Table 2). Moreover, 538 DEGs were obtained in EH vs. LH, including 163 genes with up-regulated expression and 375 genes with down-regulated expression (Fig. 2a & Supplemental Table 3). Subsequently, the heatmap indicated that the DEGs could be remarkably divided into the early-feathering and late-feathering groups (Fig. 2b). Furthermore, it was observed that 14 overlapping up-regulated genes were screened both in EC vs. LC and EH vs. LH using the Venn diagram, including DCT, SUSD3, IRF4, and NAT8L. Moreover, we noted that 9 down-regulated genes, including PTGDS, GABRA5, WNT2, EDCH1, and EDSC, were both in EC vs LC and EH vs LH (Fig. 2c & Supplemental Table 4).

### Analysis of the Function and Pathway of DEGs

**Fig. 3:**
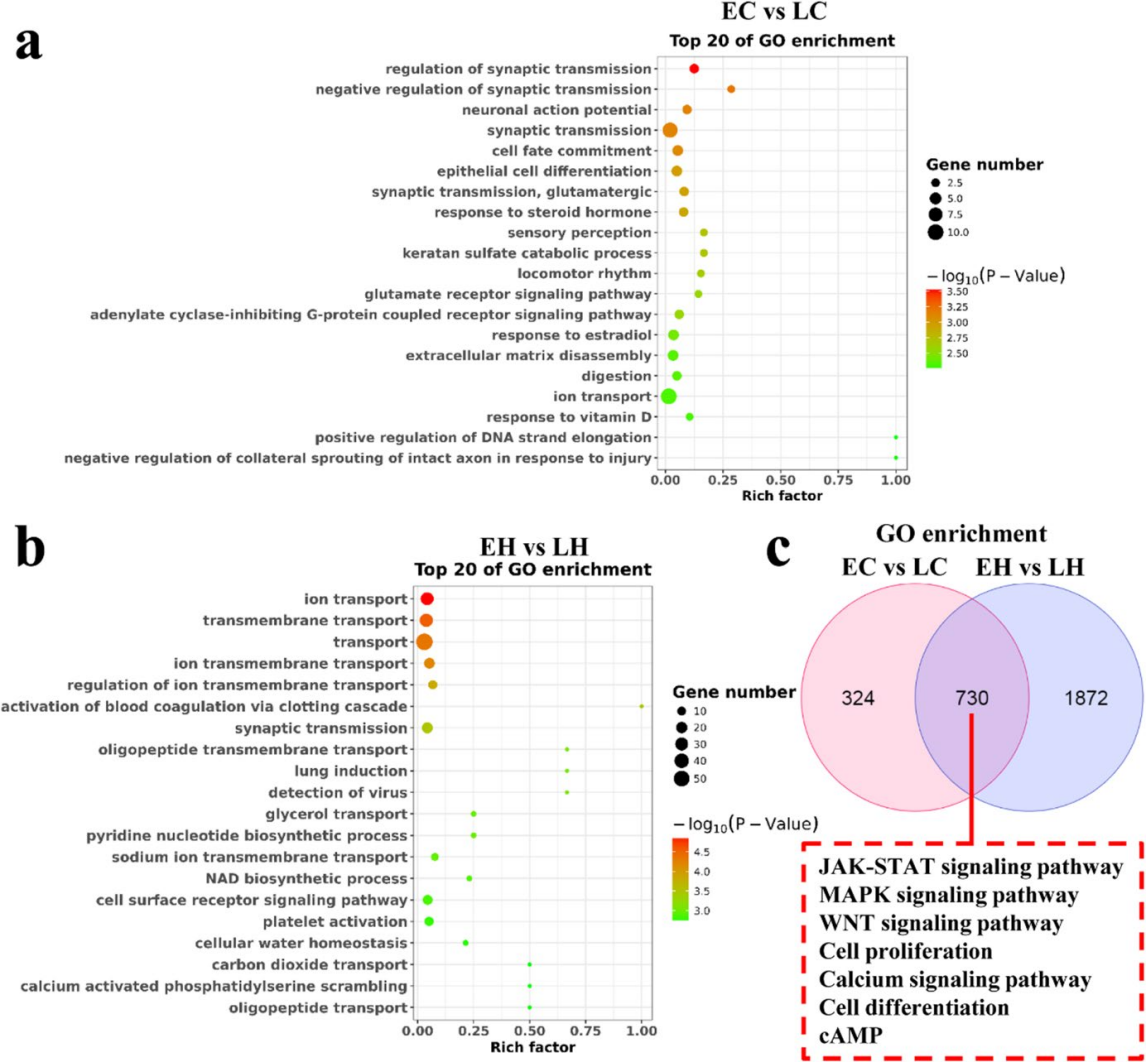
Gene Ontology (GO) enrichment analysis of the DEGs in EC vs. LC and EH vs. LH. **a** The top 20 enriched GO terms for the DEGs in EC vs. LC. **b** The top 20 enriched GO terms for the DEGs in EH vs. LH. **c** The GO enrichment of EC vs. LC and EH vs. LH was intersected

**Fig. 4:**
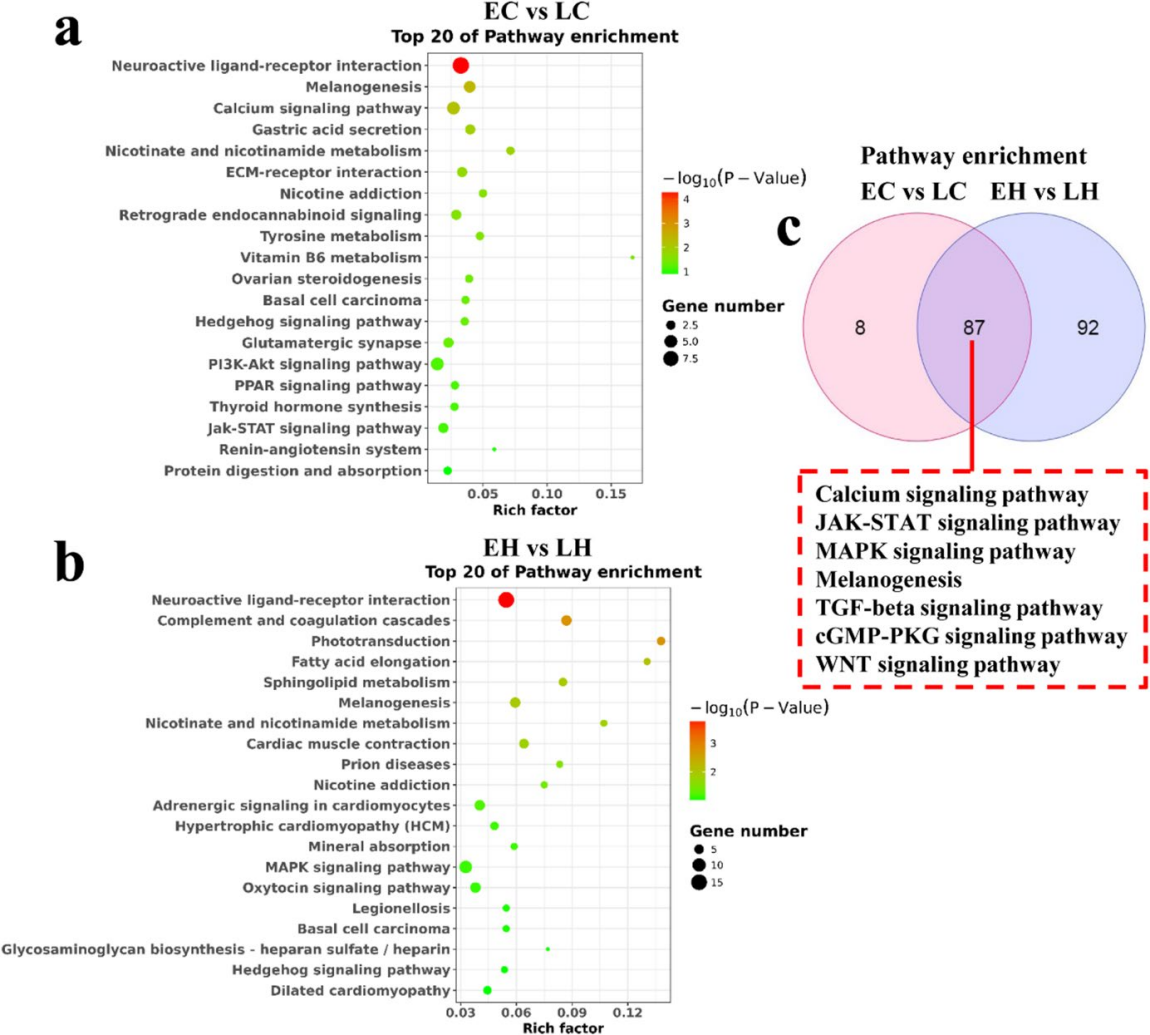
Pathway enrichment analysis of the DEGs in EC vs. LC and EH vs. LH. **a** The top 20 enriched Kyoto Encyclopedia of Genes and Genomes (KEGG) pathways for the DEGs in EC vs. LC. **b** The top 20 enriched KEGG pathways for the DEGs in EH vs. LH. **c** The KEGG pathway of EC vs. LC and EH vs. LH was intersected

To examine the function of these DEGs, GO and KEGG pathway analyses were performed to elucidate the potential role of the DEGs. GO analysis revealed that the DEGs were implicated in epithelial cell differential and extracellular matrix disassembly in EC vs. LC, as well as ion transport and cell surface receptor signaling pathway in EH vs. LH (Fig. 3a and b). KEGG analysis documented that the DEGs were associated with melanogenesis, PI3K-AKT, and JAK-STAT signaling pathways in EC vs. LC, and melanogenesis and MAPK signaling pathways in EH vs. LH (Fig. 4a and b). As shown in Fig. 3c, multiple common GO terms both in EH vs. LH and EC vs. LC were obtained using the Venn diagram software, including JAK-STAT, MAPK, and WNT signaling pathways, which have been reported to be involved in feather growth (Fig. 3c) (Tao et al. 2020; Ji et al. 2021; Feng et al. 2022). Furthermore, other feather growth-related pathways, including calcium, JAK**–**STAT, MAPK, and TGF-β signaling pathways were the common pathways in both EH vs. LH and EC vs. LC (Fig. 4c) (Li et al. 2018; Hasan et al. 2019; Tao et al. 2020; Bavananthasivam et al. 2022).

### Identification of DEPs Between Late-Feathering Chickens that Conform to Genetic Laws and Early-Feathering Chickens that do not Conform to Genetic Laws

**Fig. 5:**
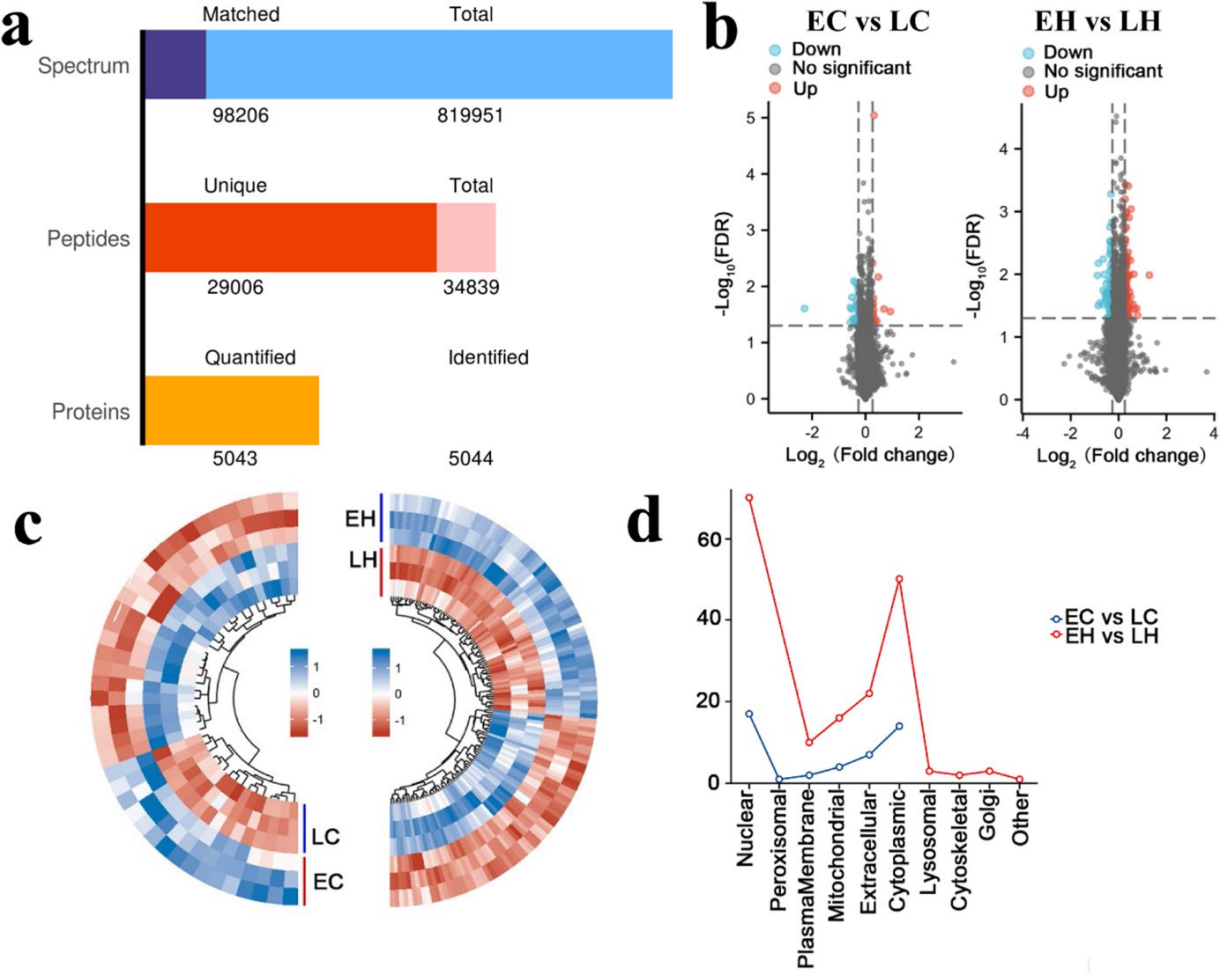
Identification of differentially expressed proteins (DEPs) in chicken obtained from mass spectrometry (MS). **a** A total of 34,839 peptides and 5,044 proteins were identified using MS (*n* = 3). **b** The volcano plot visualizes the DEPs in EC vs. LC (left panel) and EH vs. LH (right panel). **c** The hierarchical clustering heatmap of all the DEPs in EC vs. LC (left panel) and EH vs. LH (right panel). **d** Subcellular localization analysis of the DEPs

**Fig. 6:**
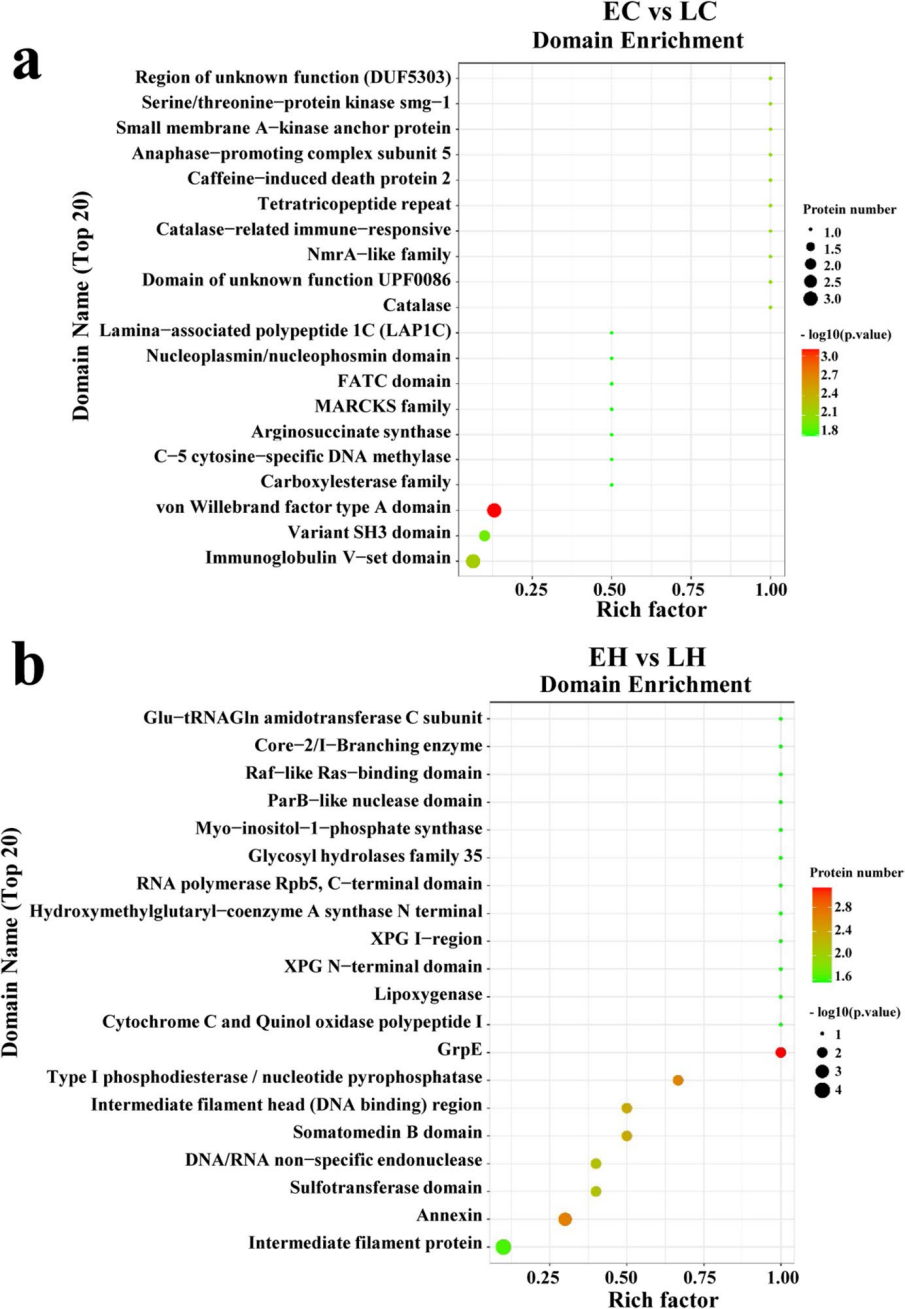
Domain enrichment analysis of DEPs in chicken. The top 20 domains for the DEPs in EC vs. LC (**a**) and EH vs. LH (**b**)

**Fig. 7:**
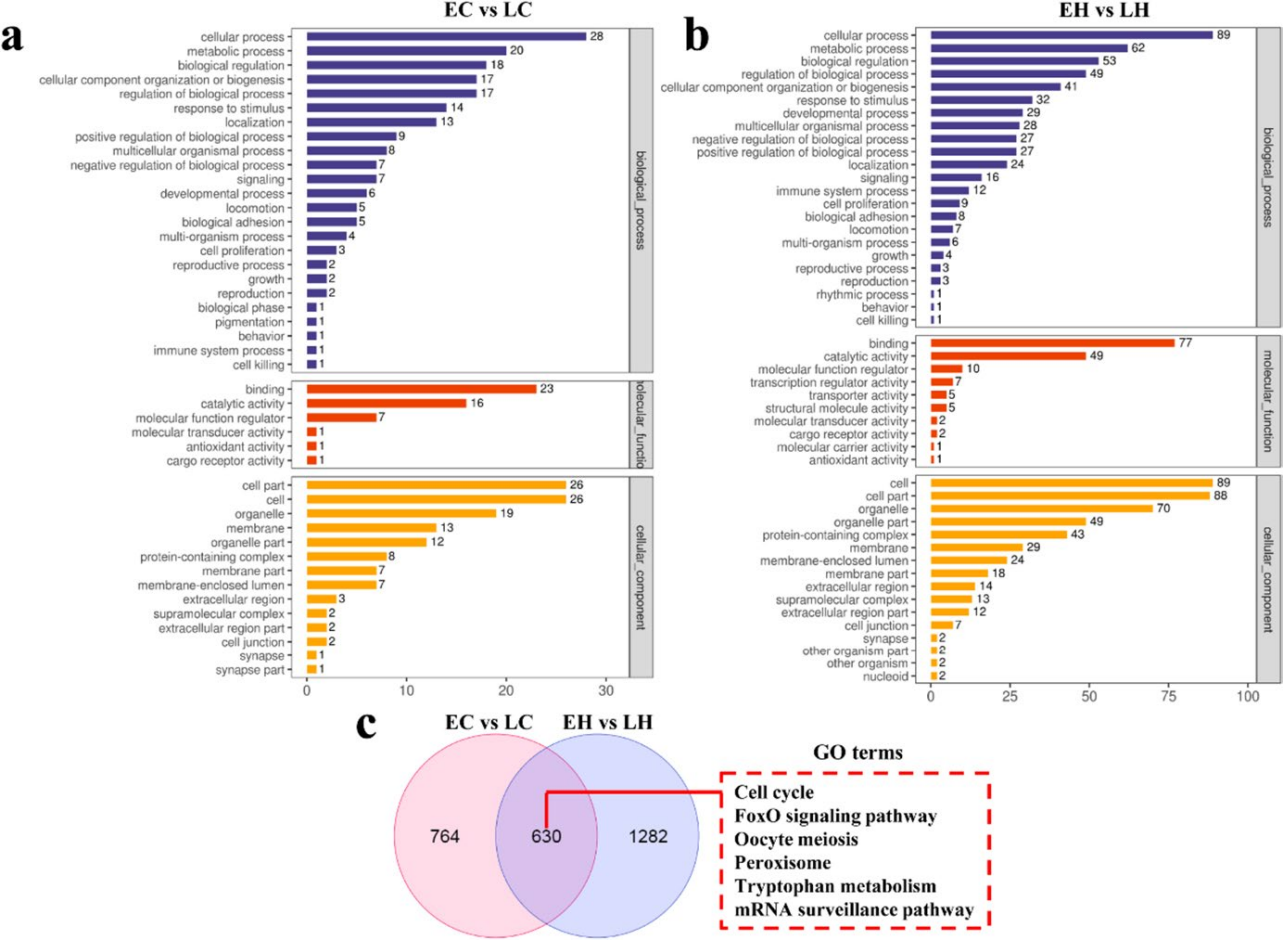
GO analysis of the DEPs in EC vs. LC and EH vs. LH. GO terms for the DEPs nearby the phenotype-related genes in EC vs. LC (**a**) and EH vs. LH (**b**). **c** The GO enrichment of EC vs. LC and EH vs. LH was intersected

**Fig. 8:**
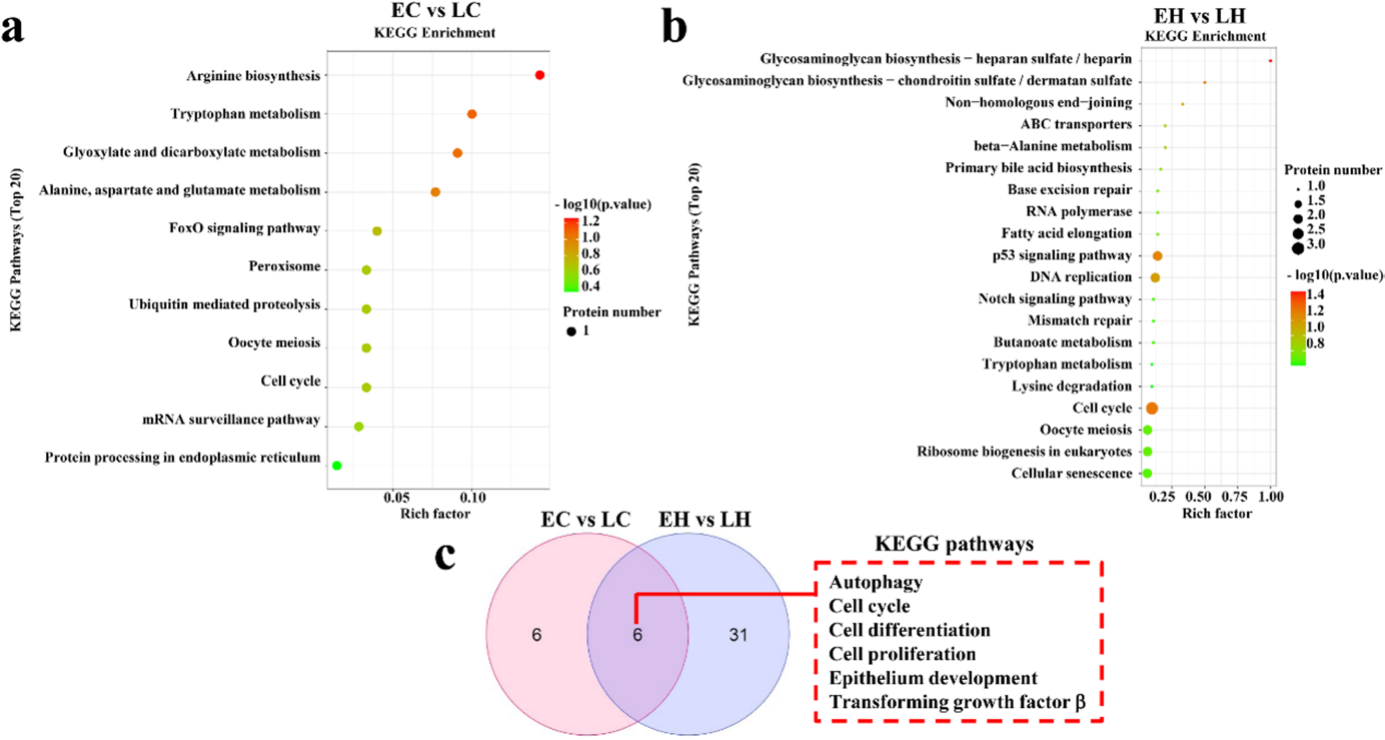
Pathway enrichment analysis of the DEPs in EC vs. LC and EH vs. LH. **a** The enriched KEGG pathways for the DEPs nearby the phenotype-related genes in EC vs. LC (**a**) and EH vs. LH (**b**). **c** The KEGG pathway of EC vs. LC and EH vs. LH was intersected

To discriminate the potential DEPs that were implicated in chicken early-feathering and late-feathering phenotypes, LC–MS/MS was used to analyze the collected sample. A total of 34,839 peptides and 5,044 proteins were screened (Fig. 5a). Differential analysis was performed on chicken samples to obtain 16 up-regulated and 25 down-regulated proteins in EC vs. LC (Fig. 5b, Supplemental Table 5). Likewise, 138 DEPs were determined in EH vs. LH, including 65 up-regulated and 73 down-regulated proteins (Fig. 5b, Supplemental Table 6). The heatmap indicated that the DEPs could be prominently divided into the early-feathering and late-feathering groups (Fig. 5c). Additionally, the subcellular localization of these proteins is depicted in Fig. 5d. Domain enrichment analysis revealed that these DEPs were implicated in multiple domains in EC vs. LC and EH vs. LH (Fig. 6a and b). To probe into the role of these DEPs, we subsequently conducted GO and KEGG analyses. It was observed that these proteins were involved in cell proliferation and growth both in EC vs. LC and EH vs. LH (Fig. 7a and b). KEGG analysis indicated that DEPs were associated with the FoxO signaling pathway, ubiquitin-mediated proteolysis, and cell cycle in EC vs. LC and correlated with DNA replication and cell cycle in EH vs. LH (Fig. 8a and b). As shown in the Venn diagrams, we noted that cell cycle, cell proliferation, and cell differentiation were common terms both in EC vs. LC and EH vs. LH (Fig. 7c). Furthermore, multiple common pathways both in EC vs. LC and EH vs. LH were obtained, including cell cycle and the FoxO signaling pathway (Fig. 8c).

### Intersection Pathway of RNA-seq and MS as Well as qRT-PCR Validation

**Fig. 9:**
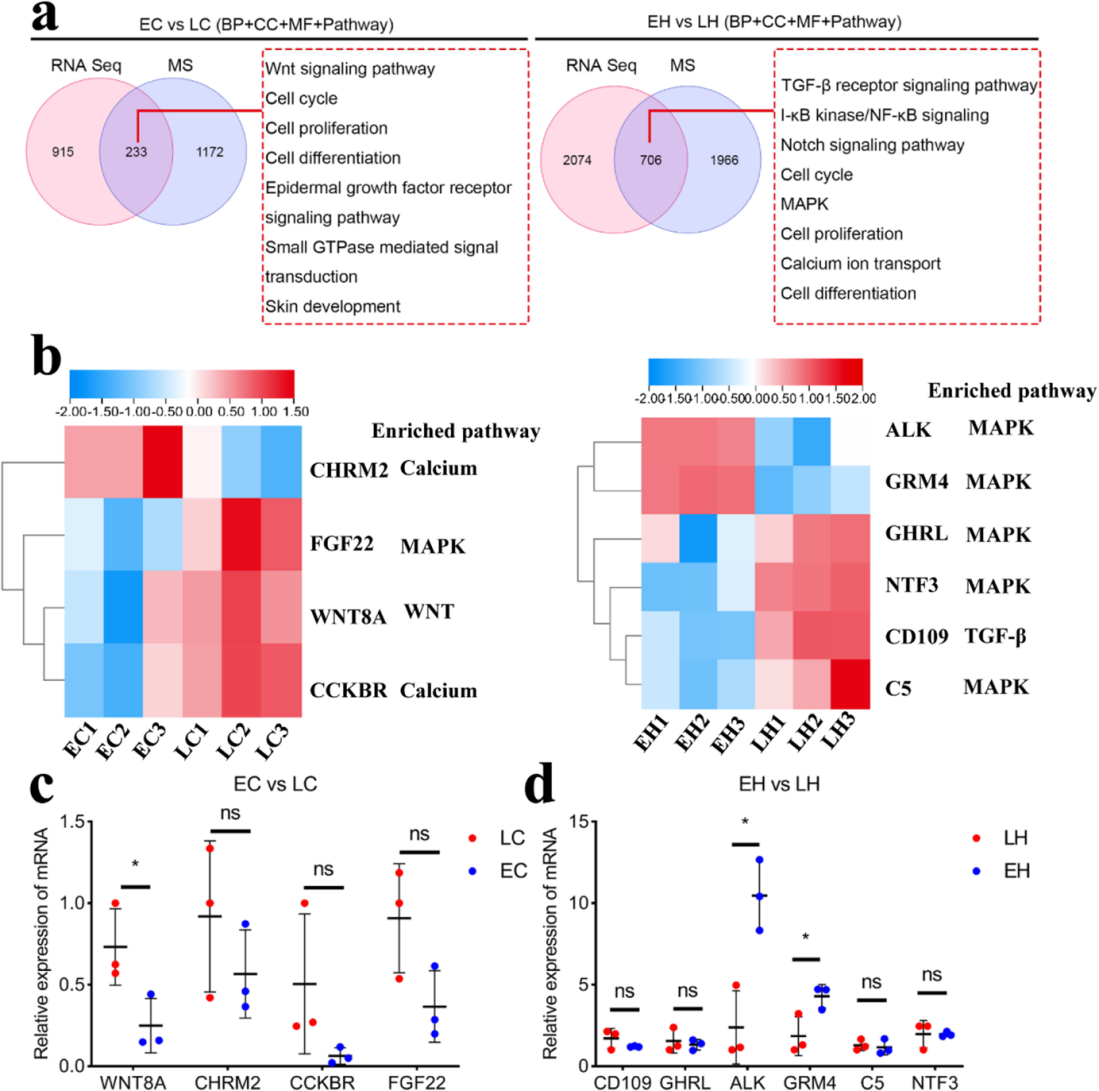
Intersection pathways of RNA-seq and MS as well as quantitative reverse transcription PCR (qRT-PCR) validation. **a** The intersection of the DEGs, BP, CC, MF, and pathways obtained from RNA-seq and MS in EC vs. LC (left panel) and EH vs. LH (right panel). **b** Heatmap of selected DEGs and enriched pathways in EC vs. LC (left panel) and EH vs. LH (right panel). **c** The WNT8A, CHRM2, CCKBR, and FGF22 mRNA expression in the indicated pathway was detected using qRT-PCR. **d** The CD109, GFRL, ALK, GRM4, C5, and NTF3 mRNA expression in the indicated pathway were verified by qRT-PCR. *ns* represents no significance. *P < 0.05

Subsequently, we took the intersection of the DEGs, biological process (BP), cell component (CC), molecular function (MF) and pathways obtained from RNA-seq and MS. Wnt, cell cycle, and cell proliferation were common factors in EC vs. LC, which may be responsible for the feathering rate phenotype (Fig. 9a). Moreover, we noted that TGF-β, MAPK signaling pathway, and calcium ion transport may be implicated in the feathering rate phenotype in EH vs. LH (Fig. 9a). DEGs enriched in the feathering rate phenotype-related pathways, including WNT, calcium, MAPK, and TGF-β signaling pathways, were selected for qRT-PCR assay (Fig. 9b). The results demonstrated that WNT8A expression was remarkably down-regulated in LC compared with EC (Fig. 9c). Additionally, ALK and GRM4 expression were significantly up-regulated in EH relative to LH (Fig. 9d).

## Discussion

In the poultry industry, feathers have shown great commercial value, including insulation to help cut down the maintenance energy needs and strengthen the poultry production yield, thereby preventing skin abrasions and infections (Clark et al. 2011; Chen et al. 2020; Ye et al. 2022). Early feathering and late feathering, which are related to sex chromosomes, are used to identify the sex of chickens; however, the feather growth rate of some chickens did not conform to the Mendelian inheritance law in chicken industry (Qiu et al. 2020). Therefore, investigating the underlying molecular mechanisms involved in the feathering rate not conforming to the Mendelian inheritance law may promote chicken-related industries. In our study, to elucidate the genes and pathways involved in the feathering rate that are not consistent with the Mendelian inheritance law, we performed RNA-seq analysis and MS between EC vs. LC and EH vs. LH. Our results identified 188 DEGs in EC vs. LC and 538 DEGs in EH vs. LH. Moreover, MS results showed 41 and 138 DEPs in EC vs. LC and EH vs. LH, respectively. Furthermore, we observed that WNT and TGF-β signaling pathways were involved in regulating the feather growth rate phenotype that was not applicable to the gene transmission mode in chickens.

The TGF-β family consists of several members, including TGF-β1 ~ 3, activins, bone morphogenetic protein, growth, and differentiation factor (Kahata et al. 2018). These proteins are expressed in the primordial buds of the feathers in the course of the late stages of chicken embryonic development (Jakowlew et al. 1994). For example, TGF-β2 plays a role in the junctional space where the epithelium and mesenchyme interact (Kahata et al. 2018). Moreover, all-trans retinoic acid could weaken the potential of hair follicle growth by means of refraining capable of proliferating and inducing apoptosis of DPCs in part via the modulation of the TGF-β2/Smad2/3 axis (Nan et al. 2020). Additionally, Bmp7, an important biomarker for epidermal organ development during the early stage of feather development of chicks, is broadly expressed in the placode epidermis and subsequently becomes localized to the forming placodes of feathers (Harris et al. 2004). In this study, we noted that the DEGs and DEPs from the combined analysis of RNA-seq and MS in EH vs. LH were significantly enriched in multiple signaling pathways, including the TGF-β signaling pathway, which furthermore suggested that TGF-β might be involved in the feather growth rate that is inconsistent with the Mendelian inheritance law in chickens.

The WNT signaling pathway plays a crucial role in embryonic development and influences the physiological phenomena of feather follicles (Feng et al. 2022). Regarding genetics, Wnt signaling is not only the initial signal for hair follicle growth but also takes part in diversified stages of morphogenesis (Hardman et al. 2015; Wang et al. 2017). Specifically, Wnt signals exert a critical regulatory effect in the evolution of the dermal papilla, cyclical changes of feather/hair follicle, and multiplication of feather follicle stem cells (Rishikaysh et al. 2014; Rognoni et al. 2016). WNT activation induces an increase in β-catenin levels; subsequently, β-catenin transfers to the nucleus, and the TCF/lymphoid-enhancing factor–β-catenin complexes serve as bipartite transcriptional activators of cyclin D1 and c-Myc (Chen et al. 2020). Additionally, Wnt/β-catenin signaling participates in the modulation of feather follicle morphogenesis and cycles (Yue et al. 2006; Ishida and Mitsui 2016). Furthermore, it has been reported that the mechanism of hair follicle regeneration for treating alopecia is related to the Wnt/β-catenin signaling pathway activation (Ito et al. 2007). In this study, we showed that the DEGs and DEPs from the combined analysis of RNA-seq and MS in EC vs. LC were significantly enriched in multiple signaling pathways, including the WNT signaling pathway, suggesting that the WNT signaling pathway might be involved in the feather growth rate in chicken.

In conclusion, our findings obtained the differential expression pattern of genes and proteins, and pathways between early-feathering chickens that abide by the genetic law and late-feathering chickens that do not follow the genetic law. The present study provides novel insights into the feather growth rate that was not in agreement with the Mendelian inheritance law in chickens.

## Supplementary Information

Below is the link to the electronic supplementary material.

Supplementary file1 (DOCX 14 KB)

Supplementary file2 (XLSX 26 KB)

Supplementary file3 (XLSX 56 KB)

Supplementary file4 (DOCX 16 KB)

Supplementary file5 (XLSX 271 KB)

Supplementary file6 (XLSX 293 KB)

## Data Availability

The datasets generated during and/or analyzed during the current study are available from the corresponding author on reasonable request.
