# Supplementary material for: Transcriptome Sequencing and Mass Spectrometry Reveal Genes Involved in the Non-mendelian Inheritance-Mediated Feather Growth Rate in Chicken
Source: Biochem Genet. 2024 Jan 27;62(5):4120–36. doi: 10.1007/s10528-023-10643-y (PMC11427531; doi:10.1007/s10528-023-10643-y)
Supplement: Supplementary file 1 — Supplementary file1 (DOCX 14 KB) [file 10528_2023_10643_MOESM1_ESM.docx]

Supplemental Table 1. Obtaining of the high-quality clean reads

| Sample | RAW reads | Clean reads | Clean per (%) | Q30 (%) | Total mapped |
| --- | --- | --- | --- | --- | --- |
| EC1 | 84977206 | 82142254 | 96.66% |  | 91.16% |
| EC2 | 96335978 | 92945348 | 96.48% |  | 91.33% |
| EC3 | 114982860 | 111334938 | 96.83% |  | 91.10% |
| EH1 | 103184602 | 99298738 | 96.23% |  | 91.30% |
| EH2 | 113366086 | 109381872 | 96.49% |  | 90.50% |
| EH3 | 119834564 | 115499494 | 96.38% |  | 91.16% |
| LC1 | 91455302 | 88531544 | 96.80% |  | 91.08% |
| LC2 | 109164960 | 104494802 | 95.72% |  | 90.92% |
| LC3 | 126802534 | 122760902 | 96.81% |  | 91.48% |
| LH1 | 91824178 | 88844956 | 96.76% |  | 90.52% |
| LH2 | 101964918 | 98824042 | 96.92% |  | 90.57% |
| LH3 | 115321506 | 1.11E+08 | 96.67% |  | 91.54% |
