# Supplementary material for: Transcriptome Sequencing and Mass Spectrometry Reveal Genes Involved in the Non-mendelian Inheritance-Mediated Feather Growth Rate in Chicken
Source: Biochem Genet. 2024 Jan 27;62(5):4120–36. doi: 10.1007/s10528-023-10643-y (PMC11427531; doi:10.1007/s10528-023-10643-y)
Supplement: Supplementary file 4 — Supplementary file4 (DOCX 16 KB) [file 10528_2023_10643_MOESM4_ESM.docx]

Supplemental Table 4. Information on overlapping genes in EH vs. LH and EC vs. LC.

| Gene | log2FC | Pvalue | Style |
| --- | --- | --- | --- |
| SCN11A (EC vs LC) | 2.174918128 | 0.003567386 | up |
| ENSGALG00000037435 (EC vs LC) | 2.02105458 | 0.000156773 | up |
| SUSD3 (EC vs LC) | 1.032352776 | 0.002082951 | up |
| DCT (EC vs LC) | 1.038481946 | 0.004264126 | up |
| CPNE7 (EC vs LC) | 1.020931635 | 0.000613965 | up |
| EYA2 (EC vs LC) | 1.640243678 | 1.95107E-06 | up |
| ENSGALG00000039659 (EC vs LC) | 1.622018358 | 4.86495E-05 | up |
| TMEM158 (EC vs LC) | 1.218022755 | 0.047593256 | up |
| CORIN (EC vs LC) | 1.041529579 | 4.36774E-09 | up |
| STK32A (EC vs LC) | 1.400375069 | 0.000350057 | up |
| GRIA4 (EC vs LC) | 3.629130201 | 5.68559E-07 | up |
| GRIK2 (EC vs LC) | 1.674340924 | 0.000425649 | up |
| IRF4 (EC vs LC) | 1.753937349 | 0.000998219 | up |
| NAT8L (EC vs LC) | 1.388907461 | 0.000299636 | up |
| ENSGALG00000014965 (EC vs LC) | -1.594773848 | 3.12389E-05 | down |
| PTGDS (EC vs LC) | -1.699173355 | 3.91109E-07 | down |
| GABRA5 (EC vs LC) | -1.73795148 | 0.000151041 | down |
| ENSGALG00000040949 (EC vs LC) | -3.713284451 | 0.018311911 | down |
| WNT2 (EC vs LC) | -1.163462064 | 0.011091741 | down |
| EDCH1 (EC vs LC) | -1.398041575 | 0.007195699 | down |
| EDSC (EC vs LC) | -1.030097877 | 0.016643569 | down |
| ENSGALG00000046632 (EC vs LC) | -1.119588874 | 0.000263231 | down |
| ENSGALG00000032318 (EC vs LC) | -1.050398172 | 0.035123405 | down |
| SCN11A (EH vs LH) | 3.932436276 | 1.53637E-08 | up |
| ENSGALG00000037435 (EH vs LH) | 1.839774638 | 0.000195062 | up |
| SUSD3 (EH vs LH) | 1.494212573 | 1.56981E-05 | up |
| DCT (EH vs LH) | 1.467598705 | 1.92101E-12 | up |
| CPNE7 (EH vs LH) | 1.478134244 | 2.85915E-05 | up |
| EYA2 (EH vs LH) | 1.497133563 | 4.85147E-07 | up |
| ENSGALG00000039659 (EH vs LH) | 1.685614585 | 2.71977E-08 | up |
| TMEM158 (EH vs LH) | 2.442015939 | 3.74344E-05 | up |
| CORIN (EH vs LH) | 1.063385685 | 1.36806E-07 | up |
| STK32A (EH vs LH) | 1.97975491 | 5.25277E-07 | up |
| GRIA4 (EH vs LH) | 2.520326351 | 0.01099129 | up |
| GRIK2 (EH vs LH) | 1.609134927 | 0.025340724 | up |
| IRF4 (EH vs LH) | 1.769647882 | 0.000108696 | up |
| NAT8L (EH vs LH) | 1.991836544 | 1.86774E-07 | up |
| ENSGALG00000014965 (EH vs LH) | -1.130367254 | 0.019793678 | down |
| PTGDS (EH vs LH) | -1.451583647 | 4.21231E-07 | down |
| GABRA5 (EH vs LH) | -1.824119466 | 6.19961E-06 | down |
| ENSGALG00000040949 (EH vs LH) | -2.33951022 | 0.04410279 | down |
| WNT2 (EH vs LH) | -1.145134345 | 0.028596342 | down |
| EDCH1 (EH vs LH) | -1.045375015 | 0.003084423 | down |
| EDSC (EH vs LH) | -1.16058031 | 3.46971E-05 | down |
| ENSGALG00000046632 (EH vs LH) | -1.651218723 | 8.24938E-08 | down |
| ENSGALG00000032318 (EH vs LH) | -1.002806545 | 0.000841531 | down |
